# Insulin as a Tool in Factitious Dysglycemia

**DOI:** 10.7759/cureus.14622

**Published:** 2021-04-22

**Authors:** Samih A Odhaib, Qussay N Almaliki, Abbas A Mansour

**Affiliations:** 1 Adult Endocrinology, Faiha Specialized Diabetes, Endocrine and Metabolism Center (FDEMC) College of Medicine, University of Basrah, Basrah, IRQ; 2 Addiction Management Center, Basrah Health Directorate, Faiha Teaching Hospital, Basrah, IRQ; 3 Diabetes and Endocrinology, Faiha Specialized Diabetes, Endocrine and Metabolism Center (FDEMC) College of Medicine, University of Basrah, Basrah, IRQ

**Keywords:** diabetes, dysglycemia, hypoglycemia, hyperglycemia, insulin, ketoacidosis, munchausen, factitious disorder

## Abstract

Factitious dysglycemia is a type of self-inflicted harm that includes deliberate attempts to induce hypo- or hyperglycemia as a sickness to gain empathy. We report the cases of three Iraqi women with different motives to induce factitious dysglycemia. Two of them had used insulin to induce hypoglycemia to have their family affection centered on them again. The third woman with type 1 diabetes mellitus intentionally missed her insulin doses to induce diabetic ketoacidosis and gain familial empathy through recurrent hospital admission, with underlying suicidal ideation. The problems with all women were discovered by a thorough history, physical examination, and with family help. They were referred to have psychiatric management. This is the first case series regarding factitious dysglycemia from Iraq.

## Introduction

Factitious dysglycemia includes an attempt to induce either hypo- or hyperglycemia by manipulating the dose of insulin or insulin secretagogues to cause sickness [1,2]. This problem is challenging to identify because most clinicians do not imagine that their patients would deliberately exacerbate their illness [3].

Factitious hypoglycemia is more common and dangerous in individuals without diabetes mellitus (DM) with some psychosocial issues, who had frequently encountered insulin in their vicinity by either a family member with DM or a relative who is a healthcare provider [4,5]. The deliberate insulin under-dosing to cause hyperglycemia and diabetic ketoacidosis (DKA) to have institutional medical care seemed inappropriately justified for some patients to fulfill dependency and self-identity needs [6].

Factitious dysglycemia should be considered a possible etiology in brittle or labile DM [5]. The management requires a long-term multidisciplinary approach with psychiatric consultation and follow-up [3,4].

Traditionally, factitious hypoglycemia can be diagnosed by low plasma glucose and C-peptide, and high serum insulin, which suggest an exogenous source of insulin. The use of new commercial assays to detect the synthetic recombinant insulin analogs is debatable, rendering the diagnosis more challenging [7]. The diagnosis of intentional missing insulin doses to induce DKA relies mainly on the high index of clinical suspicion [6,8].

We present the cases of three Iraqi women with different forms of factitious dysglycemia, different psychosocial and clinical backgrounds, and primary motives.

## Case presentation

Case 1

The first case is of an 18-year-old female in the final year in secondary school. She was the youngest daughter of a 49-year-old mother with type 2 DM (T2DM).

She was referred by her dietitian for a complaint of five attacks (after midnight hypoglycemia) in the last two weeks. However, she was disease-free with no previous relevant medical or psychiatric history. She enjoyed healthy life, was on a vegetarian diet, and visited a sports gym in the last eight months to decrease her body mass index from 32 kg/m^2^ to be 21 kg/m^2^ at presentation, with no drug therapy in any form. She did not experience any dysglycemic event during that period and complied with the monthly doctor visits.

Her older sister - a fifth-year medical student - shared the room with her, witnessed the events, and was the first-response person to diagnose hypoglycemia, which she was familiar with from her mother. She measured her sister’s capillary blood glucose level by glucometer during the events and found it in the hypoglycemic range that required emergency room (ER) referral and management.

During all events, the patient denied any intake of any culprit medication, especially insulin. In three out of the five events, she experienced persistent hypoglycemic symptoms hours after ER discharge with blood glucose confirmation.

The physical examination was non-confirmatory, with no injection marks during a full-body inspection. During the last event, a sample of her venous blood was drawn to measure the serum insulin and C-peptide the next morning, as instructed by the physician on call. Serum insulin was 32.6 µU/mL (normal value: 2.6-24.9 µU/mL, while C-peptide was 1.1 ng/mL (normal value: 0.9-4.3 ng/mL). The patient and her family were advised for careful preparedness for future events. Still, we consider insulin injection even with her denial. Her older sister was instructed to inspect for any hidden medications in their room.

Two days later, they came to the clinic after discovering a vial of premixed insulin 30/70 in a small portable cooling container, which was hidden carefully and contained three milliliters only, and a pack of insulin syringes. They were not her mother’s insulin brand or syringes. After the confrontation, the patient confessed:

“I bought this vial to burn the extra sugar in my body. I had tried to inject three units daily to see its effect and escalated the dose gradually”.

This was not convincing, given her indifference to the situation, lack of interest in further investigations, and her body language, which made us suspicious. After insisting, she stated (in tears) that:

“I injected 18 units of insulin each time to lower my sugar to draw my family's attention to me again. They constantly blamed me for the failure of a marriage proposal that came to me two months ago. They cast me away, but they were around me during these events and accepted me as am I, to do what I want to do”.

She was injecting herself at the inguinal regions, at the medial high most of the upper thigh, and the areas hidden by the underwear so that the marks could not be discovered during the former examination. Her older sister confirmed that after seeing multiple injection marks.

Case 2

The second case is of a 69-year-old retired chemistry teacher with a well-controlled T2DM for the last 11 years. She was stable on basal plus insulin regimen and extended-release metformin tablets over the previous two years, with no dysglycemic events. She had regular clinical checkups twice annually.

In the last two months, she had six hypoglycemic attacks, although she did not change her dose or the insulin type. These attacks occurred between 8:00 PM and 11:00 PM and necessitated ER management. Her clinical examination and investigations were noncontributory. Still, she was advised to decrease her insulin doses according to the readings of the self-monitoring of blood glucose.

Later on, within a week from the last attack, her 71-year-old husband, who was responsible for her insulin injection, had accidentally discovered that she deliberately injected herself with an extra dose of 15 U of rapid-acting insulin before dinner, and she pretended to miss a dose, while she was not. On confronting her, she confessed:

“I did this in the last six times to decrease my sugar to urge my kids to come to visit us and stay awhile to comfort our loneliness in this two-level gloomy home, instead of visiting us once a month or in holidays only. If they were here, this would never happen”.

Case 3

The third case is of a 32-year-old divorced housewife who lives at her parents’ home. She had uncontrolled type 1 DM (T1DM) on a basal-bolus regimen. She deliberately missed her insulin doses to induce DKA to “punish her parents for her divorce”. She was divorced after an armed tribal conflict occurred between her cousins and her ex-husband cousins on some financial issues that ended with divorcing her against her and her ex-husband's will seven years ago. She was a mother to two kids who were taken by their father’s family at court, and she was banned from seeing them according to the tribe rituals. She was diagnosed with T1DM around that period, along with severe depression and anxiety. She was managed for an attack of acute psychosis in a psychiatric ward and settled down with medication three years ago. She was diagnosed with severe lower limb sensory neuropathy and proliferative retinopathy managed by laser phototherapy and multiple intravitreal injections. She was on multiple drugs to alleviate her complaints, and she always blamed her family for her situation.

She had recurrent ER and hospital admission for the induced DKA and hyperglycemia, especially in the last year, after her ex-husband's marriage. During DKA attacks, she gained some empathy from her family, and sometimes she could meet her kids secretly with some aid from her ex-mother-in-law.

The three cases were referred to have a psychiatric evaluation with continued endocrine care through a multidisciplinary approach. Verbal and written consents were granted by the three women and the mother of the women described in the third case.

## Discussion

This paper reported cases of factitious disorders (FDs) from Iraq, where a few case reports on illness falsification for Iraqi patients have been published [9].

The three cases fulfilled the diagnostic criteria of FD, as they intentionally induced symptomatology with no external influence or incentive, with a false feeling of self-control by adopting the (sick role) [10] to replenish their devastated self-identity or to fulfill specific dependency needs and control over the past trauma. They might believe that the mandatory suffering was their way to comfort to a threshold of legalizing their suicidal thoughts through factitious behavior. The factitious behavior might grant them the power of manipulating the healthcare provider by their feigned symptomatology [6,11,12].

The most important two differential diagnoses that share a considerable resemblance with FD are suicidal attempts and malingering. Patients with DM have double the risk of depression as compared to the general population. Individuals with FD can look for therapy for their self-induced illness [13]. Malingering differs from FD in that the motive for the symptom fabrication in malingering is an external incentive and gain, which may be absent in FD [6,10]. Naqash et al. have highlighted how FD diagnosis was often associated with other mental disorders, such as depressive and anxiety disorders and some personality disorders [13].

According to the Munchausen classification, there are two distinct phenotypes of FD: Munchausen and non-Munchausen phenotypes. The Munchausen phenotype is chronic, with a characteristic antisocial and pathological lying behavior, with a social deprivation due to their ethical malpractice [8,14]. Non-Munchausen phenotype is much more typical, with a stable social environment, with no aberrant traits described for the first class. These patients are often effective in their local context [15]. The three women expressed the second phenotype.

FD is often associated with contemporary pathology to compensate for an underlying psychological deficiency, which is very difficult to ascertain [16]. Substance abuse is a recognized association in both subtypes [6] and is most commonly seen in patients with FD of Munchausen phenotype.

The accepted prevalence of FD by previous studies was 1%, which was underestimated because of the difficulty to discover the factitious practice of these patients [3,8,10,16]. The health providers need a high index of suspicion given the lack of precise laboratory tests for biochemical diagnosis. The diagnosis of FD relies on thorough history from all family members, with a special concern about the social history, searching for any psychological cues about possible FD [17].

The choice of the strategy to approach and manage FD cases, as shown in the described cases, might take different therapeutic approaches, in which confrontational and non-confrontational strategies were described, and a non-confrontational style can be chosen based on a multidisciplinary evaluation [18].

The management plan should be objective to diagnose the fabricated illness, not its motive, or why the patient had such deceiving behavior [10,19]. It is imperative to remember that individuals with genuine medical problems may also falsify symptoms and that individuals with FD are suffering and need expert care [8,16]. Patients with Munchausen's syndrome may have organic disease like anybody else [16].

The different insulin-measuring platforms are not useful in detecting the highly complex insulin analog because of low cross-reactivity [7,17,20].

The medical management of factitious dysglycemia with the aid of the psychiatric evaluation is essential for confronting the patient and assessing the future management plan, although the long-term prognosis is unpredictable [17] given the lack of readiness to engage in long-term psychotherapy [6,12,16].

Genuine illnesses such as brittle DM may have a psychiatric background that contributes to its pathogenesis, considering FD as an etiology. This may explain the partial recovery of brittle DM in about 50% of cases after treating the underlying causes, while others may need long-term psychotherapy [19].

Patients without DM are keen to hide their puncture sites, syringes, and their insulin even during an exhaustive search, which may raise some ethical complaints on some occasions even with their consent [1]. That was the condition in the first described case. The other two cases were diabetic and already on multiple insulin injections.

Eventually, emotional instability, the distorted subjective impact of the problems of dysglycemia on their life, the indifference regarding the consequences of their action, and the deception to be viewed as sick or impaired to satisfy a psychological need were the warning signs for the clinicians to diagnose FD in cases 1 and 3.

For case 2, the noncontributory medical history and early discovery of insulin abuse to induce hypoglycemia decreased the need for further investigations and reduced the referral time.

## Conclusions

Although factitious dysglycemia through insulin misuse is infrequently encountered in the medical practice in individuals with or without DM, it might be considered a sign of a hidden severe psychiatric illness. A solid clinical index of suspicion is needed by the healthcare providers for diagnosis and possible psychiatric referral for management.
